# Schwannoma in the Rectum: A Case Report

**DOI:** 10.7759/cureus.61571

**Published:** 2024-06-03

**Authors:** Mikayla Hobbs, Ryan Denis, Martin Felix, Marianna Zeichen

**Affiliations:** 1 General Surgery, St. George's University School of Medicine, St. George, GRD; 2 Colorectal Surgery, Jackson Memorial Hospital, Miami, USA

**Keywords:** gastrointestinal oncology, lower gi or colorectal surgery, colorectal neoplasm, gastrointestinal schwannoma, rectal schwannoma

## Abstract

A woman in her 60s with a past medical history of recurrent *Helicobacter pylori* (*H*. *pylori*) presented for surgical consultation after a colonoscopy revealed a mass in the rectum. Preoperative biopsy revealed mucosal excrescence with no dysplasia or malignant changes. The final pathology showed a solid, submucosal rectal mass that was positive for SOX10 and S100 on immunohistochemistry, supporting our diagnosis of Schwannoma. This case emphasizes the importance of considering schwannomas in the differential diagnosis of patients presenting with a rectal mass no matter how rare it may be.

## Introduction

Schwannoma tumors are rare and often benign on presentation with the potential to become cancerous, so the need for early diagnosis is critical. Schwannomas originate from Schwann cells, which form the neuronal sheath and play a crucial role in maintaining the peripheral nervous system (PNS). These tumors can grow in many parts of the body such as the arms, legs, face, and brain. They uncommonly form in the gastrointestinal tract (GIT). Among all GIT schwannomas, those found in the colon and especially the rectum are extremely rare, with only 28 cases reported in the year 2019 [1]. Schwannomas can occur in different parts of the GIT, including the stomach (incidence of 83%), the small bowel (incidence of 12%), and the colon and rectum (incidence of 2-6%). Similar rates of occurrence have also been observed in men and women, with a mean age of 60-65 years old [2]. Typically, these tumors are discovered incidentally by imaging studies, endoscopy, or colonoscopy. Occasionally, when patients present with symptoms, they tend to have a non-specific clinical presentation of abdominal pain, rectal bleeding, melena, tenesmus, etc., similar to any other GIT tumor [1].

Differentiating Schwannomas from other mesenchymal tumors like gastrointestinal stromal tumors (GISTs) or adenocarcinomas is also very important, as they may present similarly. Endoscopically, these tumors present with smooth mucosa or mucosal ulcerations and usually give limited information for differentiation making preoperative diagnosis difficult. To confirm a schwannoma, diagnosis via immunohistochemical analysis will need to show tumor cells carrying a positive status for the distinctive S-100 protein [3]. Overall the prognosis of schwannomas in the GIT is good due to most having low malignant potential and benign status. A complete surgical resection with negative margins will be the best treatment option, significantly lowering the chances of recurrence [1-3]. Again, with the very limited cases of schwannoma in the rectum, our case report aims to increase the awareness of these tumors being considered as a differential diagnosis.

## Case presentation

**Figure 1:**
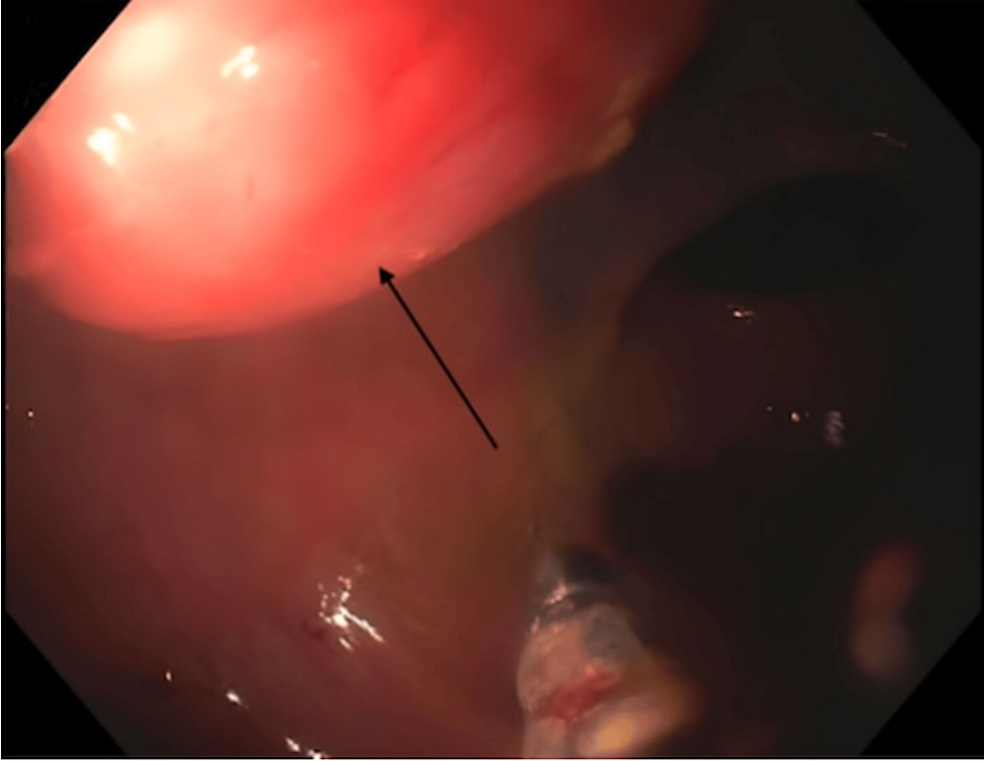
Colonoscopy image revealing a submucosal rectal mass

**Figure 2:**
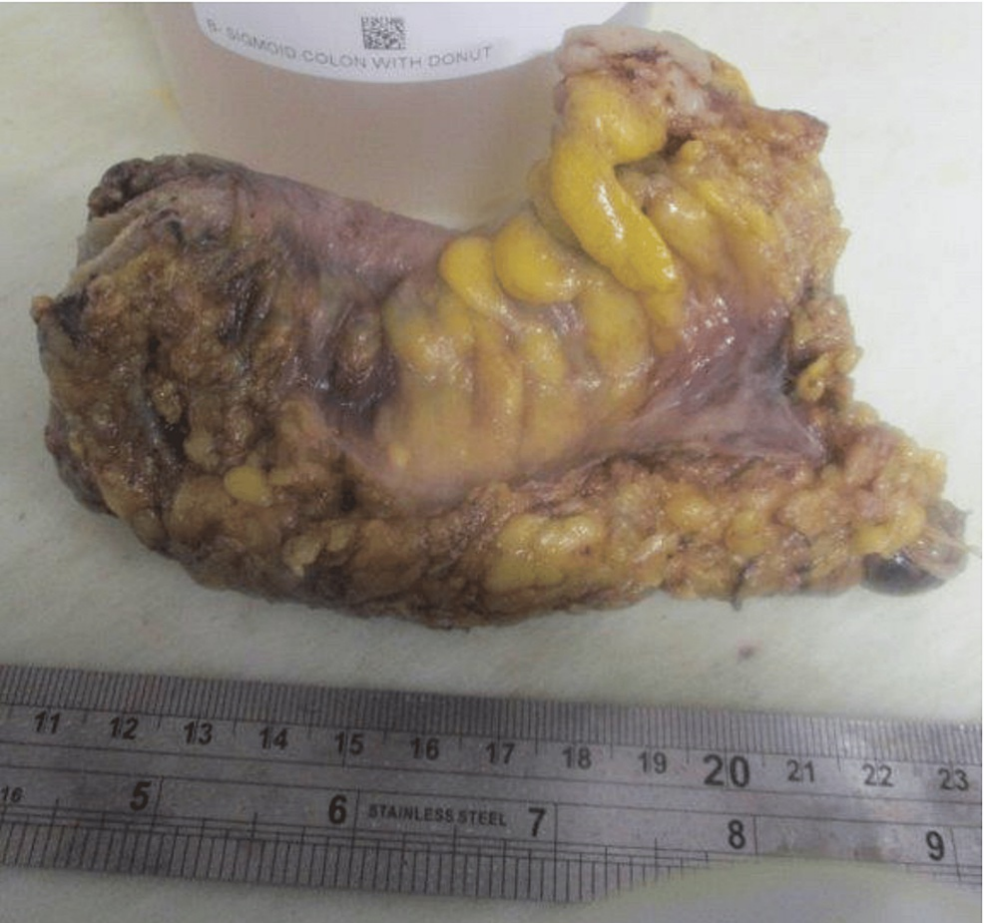
Full surgical specimen following resection

**Figure 3:**
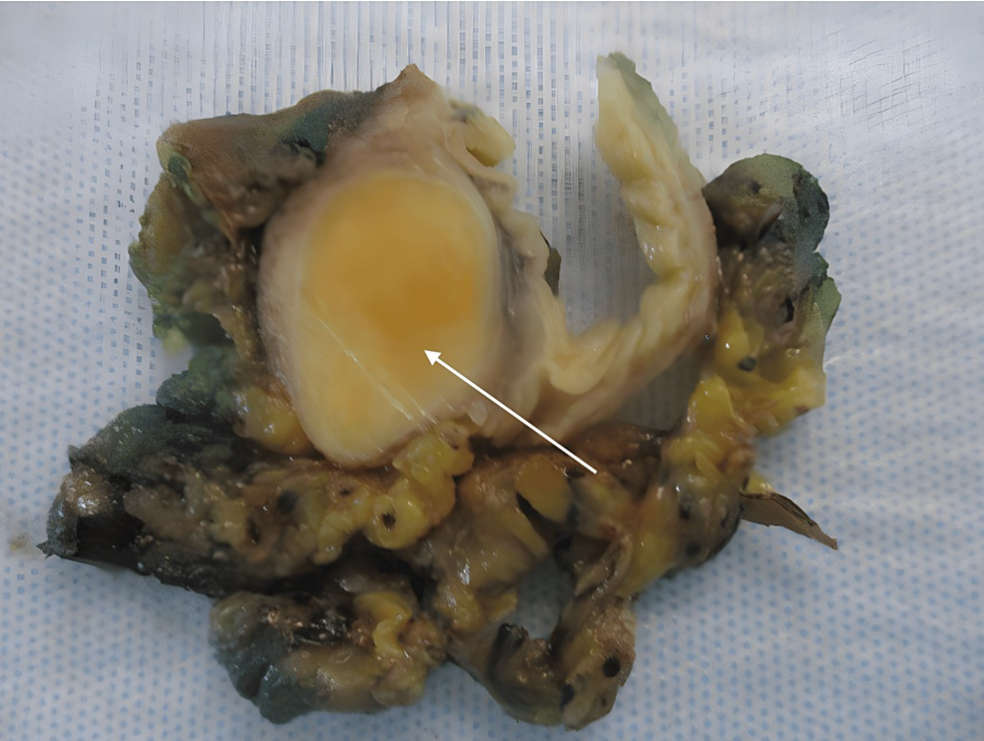
Gross image of the rectal mass post-resection

**Figure 4:**
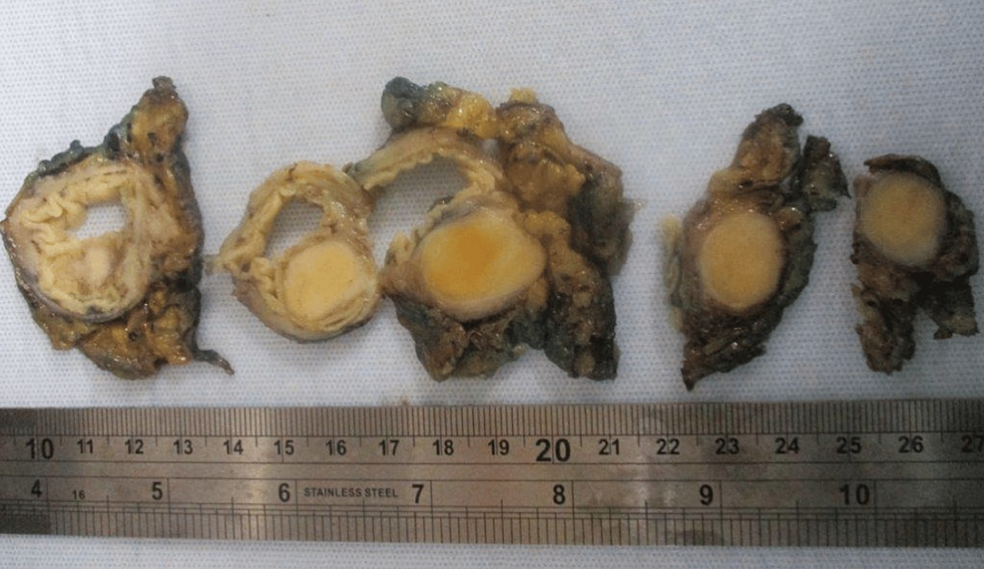
Transections of the rectal mass post-resection

**Figure 5:**
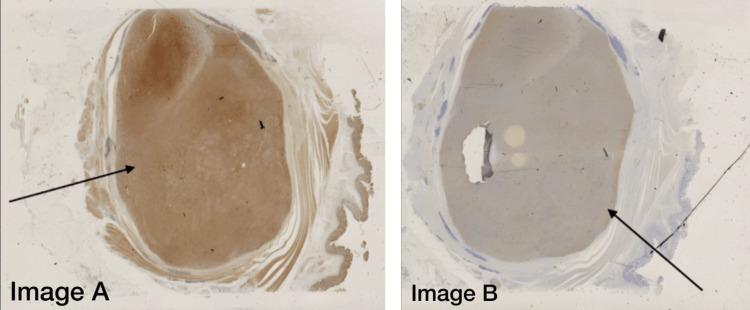
Immunohistochemistry slides of the resected mass Image A: Immunohistochemistry representing positive membrane staining for SOX10 Image B: Immunohistochemistry representing positive membrane staining for S100 (sensitive and specific for Schwannoma)

A 64-year-old woman with a past medical history of recurrent *Helicobacter pylori (H. pylori) *presented for a routine colonoscopy that revealed a 20 mm submucosal nonbleeding mass in the rectum (Figure 1). At the time of the colonoscopy, the patient denied nausea, vomiting, and diarrhea but did endorse chronic constipation. Magnetic resonance imaging (MRI) was performed, revealing a 2.1 cm mid-rectal mass, arising deep into the mucosal layer, suggesting a possible gastrointestinal stromal tumor. Several biopsies of the mass were taken, a tattoo was performed, and the patient was referred for surgical consultation. The surgical team recommended robotic lower anterior resection. While in surgery, the tattoo was visualized in the rectum by the surgical team, and a robotic lower anterior resection was performed (Figure 2). Pathology of the resected rectum showed a 2.2 x 2.2 x 1.8 cm tan firm, solid submucosal mass involving a portion of the circumference of the rectum as well as the muscularis propria (Figures 3-4). Immunohistochemistry was performed revealing tumor cells positive for SOX10 and S100 (Figure 5) and negative for desmin, H-Caldesmon, CD117, and DOG-1, supporting our diagnosis of cellular schwannoma. Fifteen lymph nodes were also removed in which all were benign. No further treatment was necessary for this patient as the tumor was benign. The patient has been following up outpatient with the surgical team with no complications.

## Discussion

Schwannomas are usually solitary, benign, well-circumscribed masses of the peripheral nerves that appear smooth and nodular in appearance, often covered by a fibrous capsule composed of epineurium and other nerve fibers [4]. Microscopically, they are characterized by fascicles of Schwann cells alternating between highly cellular, spindle cell morphology (Antoni A pattern) merging with less cellular, loosely textured, microcystic areas (Antoni B pattern), as well as intermittent areas of palisading, parallel nuclear arrays (Verocay bodies) [5]. Schwannomas can present in just about any part of the body; however, they are typically located in the upper limbs, followed by the head, trunk, and flexor surfaces, and more rarely, the GIT [6]. Of those presenting in the GIT, they most commonly occur in the stomach. Primary Schwannomas of the rectum, which are not associated with other syndromes, are extremely rare [7]. Our patient presented with a primary mass in the rectum with no other medical history, making this location of Schwannoma considerably rarer with only a few documented cases. This condition can recur, and, although uncommon, may become malignant if left untreated. Therefore, it is paramount to distinguish it from other intestinal mesenchymal neoplasms, such as smooth muscle tumors, neurofibromas, and GISTs, to accurately plan therapeutic strategies [8].

The pathogenesis of Schwannomas is generally sporadic, although some appear in specific syndromes, such as neurofibromatosis type 2, schwannomatosis, and Carney complex, indicating a possible genetic etiology [6]. Diagnosis of gastrointestinal presenting Schwannomas relies on the use of both noninvasive (MRI and computed tomography (CT) scans) and invasive imaging (endoscopy, colonoscopy, and barium enema) with confirmation by biopsy. The use of the former modalities does not allow for differentiation from other intestinal neoplasms. Biopsy with immunohistochemical testing is the most accurate mode for diagnosis. Immunohistochemistry illustrating tumor cells positive for S-100 and negative for CD117 is important for differentiating intestinal schwannomas from other mesenchymal neoplasms, which was the case in our patient [9].

Schwannomas presenting in the GIT are generally asymptomatic and incidentally found on routine endoscopy, as was the case in our patient. Rectal Schwannomas may present with similar features to other masses with signs of obstruction, bleeding, or tenesmus [2]. Generally, this type of mass is considered benign; however, there is a risk of local recurrence and malignancy. The degree of aggressiveness depends on both the Ki-67 and mitotic indexes, with the Ki-67 index being the indicator of malignancy. A value of more than 5% correlates with increased tumor aggressiveness while a value of 10% is considered malignant [2]. Tumors with high mitotic activity or size larger than five centimeters are associated with a higher risk of metastasis and/or recurrence [2]. Complete surgical resection with negative margins is the mainstay mode of treatment; however, removal of rectal presenting masses may result in significant complications such as anal incontinence and/or perioperative bleeding. One study found that performing a perianal inter-sphincteric approach, as opposed to traditional approaches, such as trans-sphincteric, trans-sacral, or trans-anorectal excisions, resulted in decreased perioperative bleeding and anal incontinence as well as less contamination [10]. This method could be influential in future cases to decrease postoperative complications.

## Conclusions

Schwannomas are rare benign tumors derived from Schwann cells and appear nodular and smooth in appearance. Schwannomas can be found hypothetically anywhere in the body where Schwann cells are found and are rarely found in the rectum with an incidence of 2-6%. Although benign, Schwannomas may become malignant so when diagnosed, resection is usually the plan of care. Considering our patient's complaint of constipation and a mass found in the rectum with an inconclusive preoperative biopsy, surgical resection was performed. The pathology post-resection with immunohistochemistry showed cells positive for S-100 leading to the diagnosis of a Schwannoma. Typically, GIT Schwannomas have a great prognosis with a low chance of recurrence requiring routine follow-up care. Our case demonstrates a rare tumor in an even more uncommon location and will hopefully be considered as a differential diagnosis for tumors presenting in the rectum.
